# Increase in lipid portion of Phycomyces blakesleeanus biomass induced by vanadate uptake and accumulation

**DOI:** 10.1099/mic.0.001615

**Published:** 2025-09-24

**Authors:** Jovana M. Lukičić, Milena Dimitrijević, Milan Žižić, Željka Višnjić Jeftić, Miroslav Živić, Tijana Cvetić Antić, Marina Stanić

**Affiliations:** 1 Faculty of Biology, University of Belgrade, 11158 Belgrade, Serbia; 2 Institute for Multidisciplinary Research, National Institute of Republic of Serbia, University of Belgrade, 11030 Belgrade, Serbia

**Keywords:** Fourier Transform Infrared (FTIR) spectroscopy, fungi, lipid content, vanadium uptake

## Abstract

Fungi are ubiquitous micro-organisms involved in various environmental processes, with a particularly important role in the transformation of metals and minerals, bioremediation and biomining. Filamentous fungus *Phycomyces blakesleeanus* is an interesting model for investigating the interaction of fungi with various ecological factors, such as heavy metals, due to the ease of its cultivation and fast growth. The present study deals with the interaction of increasing vanadate [V(V)] concentrations with the mycelium of *P. blakesleeanus* in three distinct growth phases: mid-exponential, late exponential and stationary phase. Mid- and late-exponential phase mycelium had a V content of nearly 1% after 24 h incubation with 10 mM V(V), and the uptake of V(V) was accompanied by increased phosphorus uptake with both 5 and 10 mM V(V). Fourier Transform Infrared spectroscopy showed the increase of lipid portion in biomass compared to proteins and carbohydrates mainly with ageing, but also with vanadate treatment. *P. blakesleeanus* is tolerant to high V(V) concentrations, and this study suggests its potential as V accumulator. In addition, the increase in lipid content calls for a closer examination of lipid content and fatty acid composition after V(V) treatment and determination of their potential industrial utilization.

## Graphical abstract

Mycelia of *Phycomyces blakesleeanus* were incubated for 24 h in V(V) at three different growth stages: mid-exponential (20 h), late exponential/early stationary (36 h) and stationary (56 h). The uptake of V(V) was highest in the mid-exponential stage, while a significant increase in the lipid/amide I ratio was observed in both the mid-exponential and late exponential/early stationary stages.

## Introduction

Interactions of micro-organisms with transition metals is a topic that has been tremendously represented in literature in the last few decades. Microbes can change the physical and chemical states of metals, while metals affect microbial growth, survival and activity [1] as they are directly or indirectly involved in all aspects of growth and metabolism [2]. Fungi play an important role in various environmental processes through their participation in biogeochemical cycles of elements, transformations of metals and metalloids, bioremediation and the formation of biominerals [3]. Vanadium (V), the 22nd most abundant element in the Earth’s crust [4], is essential for many eukaryotic organisms at micromolar concentrations [5], and some fungi are known to contain vanadate-dependent enzymes [6], while some of them are able to accumulate it [3,6,8], making them one of the pathways through which vanadium enters the ecosystem [9]. In lower concentrations, vanadium is viewed as a promising medical agent due to its insulin mimetic effect [10] and potential as an anti-cancer drug [11], as well as an antiviral and antibacterial agent [12]. However, as ‘the dose makes the poison’, the increasing presence of V in the environment raises concerns about its neurotoxicity, gastrointestinal problems, emphysema and pneumonia [13]. The effects depend primarily on the oxidation state, chemical form, dose and duration of exposure [14], with the most common oxidation forms being vanadate [V(V)] and vanadyl [V(IV)]. Changing industrial practices increases the rate at which vanadium is deposited into the environment, which calls for ecologically clean methods of bioremediation.

Many fungi can tolerate V and mobilize it from minerals or even accumulate it [3,8], which led to consideration of their possible role in bioremediation. On the other hand, metabolic transformations of V in fungi candidates them as potential bio-factories of V complexes with desirable pharmacokinetic properties. Finally, fungi are rich in various high-value metabolites such as lipids, proteins, pigments, polyphosphates and chitin/chitosan [15], the content of which can be increased by the application of stressors, and V is a redox-active metal.

The fungus *Phycomyces blakesleeanus* was selected for this research of V uptake and metabolic response due to its well-studied physiological response to acute V exposure [16,18]. It grows successfully on vanadate-free medium, and no information on the presence of vanadium-dependent enzymes was found when reviewing the available literature. From Lukičić *et al*. [19], the mycelium of this fungus is known to tolerate high concentrations of V (10 mM) for at least up to 5 h of exposure. Its fast growth and short life cycle make it a good candidate for potential industrial application. Here, the mycelium of *P. blakesleeanus* was exposed to increasing vanadate V(V) concentrations at three different growth stages (mid-exponential, late exponential and stationary stage) for 24 h to determine its uptake potential and the effects of V(V) on the content of major biomolecules such as lipids, proteins and carbohydrates.

## Methods

### Organism, growth conditions and V(V) treatment

**Fig. 1.**
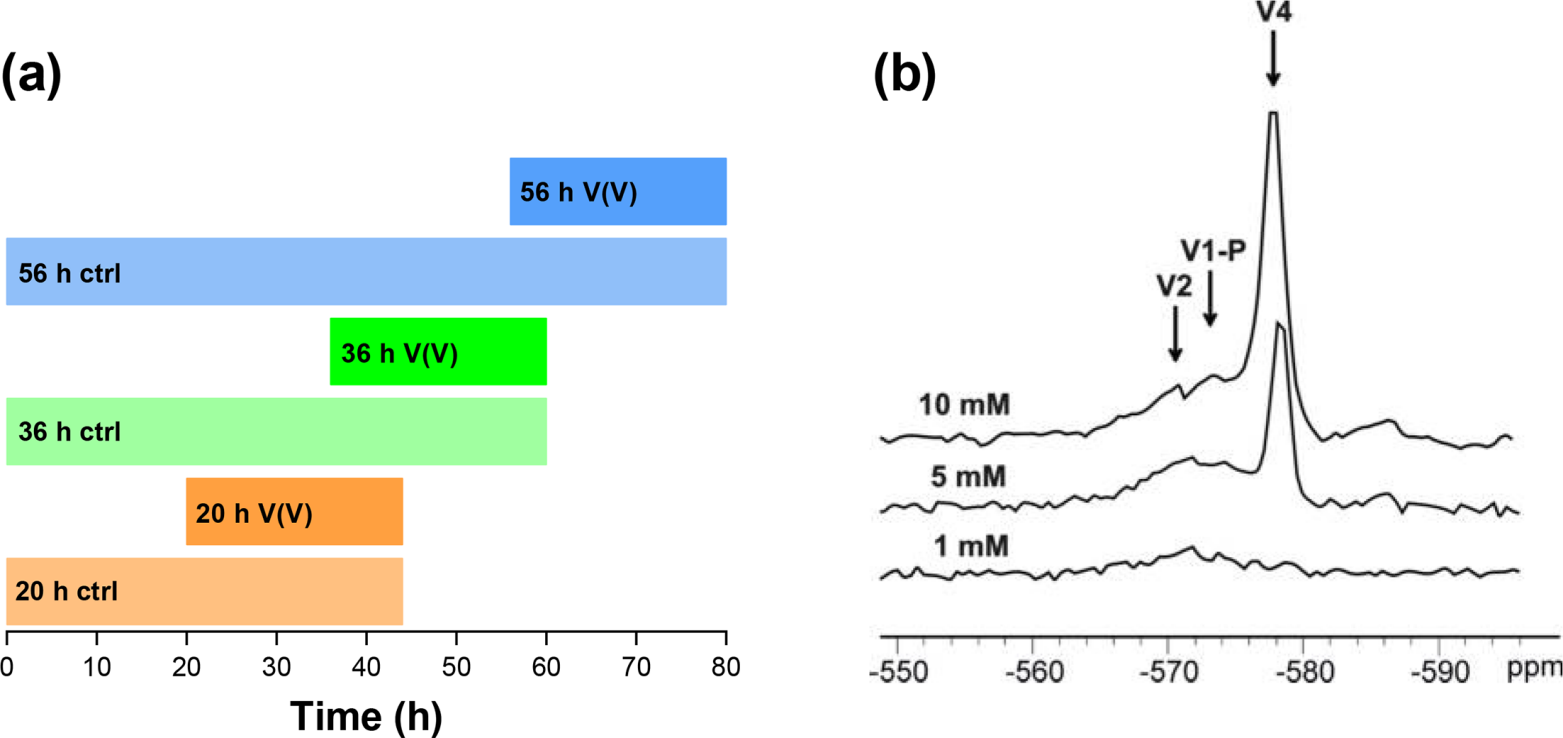
(**a**) Experimental timeline: *P. blakesleeanus* mycelia were treated with increasing V(V) concentrations for 24 h at different growth stages: mid-exponential (20 h), late exponential (36 h) and stationary (56 h). Controls were assayed at the end of the treatment period, so their actual ages were 44, 60 and 80 h. (**b**) Vanadate speciation at different concentrations in growth medium at constant pH 6.7.

All experiments were performed on the mycelium of WT fungus *P. blakesleeanus* (Burgeff) [NRRL 1555(-)]. The stock solution of 200 mM sodium orthovanadate (Na_3_VO_4_) (Sigma-Aldrich, Taufkirchen, Germany) was prepared according to the method of Gordon [20]. Spores (10^5^ spores ml^−1^) were heat activated for 15 min at 48 °C and inoculated into Sutters liquid standard minimal medium (SIV) [21], with pH modified from 4.5 to 6.7, in 100 ml Erlenmeyer flasks filled to half capacity for better gas exchange, shaken at 22 °C at 120 r.p.m. on a digital orbital shaker, under continuous white fluorescent light of 10 W m^−2^. Mycelium was grown for 20 (early to mid-exponential phase), 36 (late exponential phase) and 56 h (stationary phase) [19], collected by vacuum filtration and transferred to fresh medium containing 1, 5 or 10 mM Na_3_VO_4_ for 24 h. Addition of vanadate stock solution significantly raises the pH of the medium. To avoid the effects of pH on mycelial metabolic response, it was necessary to transfer mycelium to pre-prepared medium containing appropriate V(V) concentrations and with adjusted pH. The controls were handled as the treated samples, i.e. transferred to fresh medium pH 6.7, except without V(V) incubation, making their real age at the time of assay 44, 60 and 80 h (Fig. 1a). For simplicity, they will be referred to as 20+24, 36+24 and 56+24 h controls.

### ^51^V NMR spectroscopy: V(V) speciation

The species of vanadate present in the medium was determined by using ^51^V NMR spectroscopy. Sodium orthovanadate was added to the growth medium in concentrations of 1, 5 and 10 mM [21], and the pH was further adjusted to 6.7 with KOH. The measurements were performed on an Apollo upgrade (Tecmag, USA), a Bruker MSL 400 (Germany) spectrometer operating at 105.169 MHz for ^51^V, using a Bruker static solid-state probe, with the sample in a horizontal position. The pulse duration was 20 µs (45°), and the spectra recording duration was ~3.6 min. Chemical shifts are given relative to external 1 M sodium metavanadate, pH 12, which produces two signals in aqueous solution: the monomer at −535.7 p.p.m. (used as an auxiliary reference) and the dimer at −560.4 p.p.m., relative to VOCl_3_ [12].

### ICP-OES analysis: V and P uptake

Inductively Coupled Plasma-Optical Emission Spectroscopy (ICP-OES) analysis was used to determine V and P content in the mycelium of *P. blakesleeanus*. After the treatments, the samples were centrifuged at 4,300 ***g*** for 5 min at 4 °C, washed twice with distilled water and oven-dried at 60 °C for 48 h. The dried mycelium (~500 mg) was digested in a microwave digester (ETHOS EASI, Milestone, Italy), with 2 ml of 30% hydrogen peroxide (Merck, Germany) and 8 ml of 65% nitric acid (Merck, Germany) at 180 °C. Analytical blank samples (four in total) were prepared to resolve the potential presence of analysed elements in the reagents used. After cooling at room temperature, digested samples were diluted with distilled water to a total volume of 20 ml. The analysis was carried out by inductively coupled plasma spectrometer (ICP-OES, Avio 200, Perkin Elmer) at the wavelength lines of 292.464 nm for V and 213.617 nm for P.

### Intracellular ROS determination

Reactive oxygen species (ROS) were determined by following the hydrolysis of dichlorodihydrofluorescein diacetate (DCFH-DA) to DCFH-carboxylate anion, which is readily oxidized to highly fluorescent dichlorofluorescein (DCF) in the presence of various oxidative agents [22]. For this assay, the fungus was grown as described, and after a 24 h treatment with V(V), the mycelium was collected and diluted with water to an OD_750_ of ~0.5. The diluted samples were washed twice and incubated with DCFH-DA for 1 h in the dark at room temperature. The dye was dissolved in 96% ethanol and added to the samples to a final concentration of 20 µM. The samples were further diluted with water when the fluorescence reached saturation, and 200 µl of each was added to clear bottom black microtiter plates. DCF fluorescence at ex/em 485/530 nm was measured using a Tecan Infinite M Nano microplate reader (Tecan Group Ltd., Maennedorf, Switzerland). The results are presented as DCF fluorescence intensity of the sample, taking dilution into account, and as the fluorescence ratio of treatment and control. When calculating fluorescence ratios, fluorescence intensities of V(V) treated mycelia for 24 h were normalized to controls of appropriate growth stages (20+24, 36+24 and 56+24 h). The fluorescence intensity of DCF is directly related to the presence of oxidative species, mainly H_2_O_2_, in the samples.

### ATR-FTIR spectroscopy

Attenuated Total Reflectance with Fourier Transform Infrared (ATR-FTIR) spectroscopy (SpectrumTwo, PerkinElmer, Waltham, MA, USA) was used to analyse metabolic fingerprints of the fungal mycelia. For the FTIR analysis, the incubation period of 5 h (with appropriate controls) was tested alongside 24 h incubation with V(V). After the treatments, the mycelium was washed twice with H_2_O, snap-frozen in liquid nitrogen and freeze-dried. The dried biomass was homogenized with 1 mm stainless steel beads for 10 min (Mixer Mill MM400, Retsch, GmbH) at 30 Hz. Spectroscopic measurements were performed in transmission mode using a flat-tip pressure arm with a gauge force of 95–100. The spectra were recorded in the wavenumber range from 4,000 to 400 cm⁻¹, with a resolution of 1 cm⁻¹ and 16 accumulations per sample. All spectra were automatically corrected for water vapour and the baseline using the correction algorithms available in the Spectrum 10 software. Spectra analysis was performed using Quasar 1.10.2 software.

### Statistical analysis

The statistical significance of differences was analysed using a one-way ANOVA with a significance level of 0.05. Pairwise comparisons made with the Holm-Sidak test are indicated by the letters above the bars (a, b, c). Differences between treatments and controls in the FTIR analysis are marked with black asterisks. GraphPad Prism 6.01 was used for statistical analysis and graph generation. The principal component analysis (PCA) of the correlation matrix was used to summarize the interrelations of developmental and V(V) effects on the content of lipids and carbohydrates in the mycelium. PCA was computed using XLSTAT (version 7.5.2) software (Addinsoft).

## Results and discussion

### Speciation and forms of V(V) in the medium

The speciation of vanadate in aqueous solution is the cumulative result of several factors, of which concentration, pH and ionic strength are considered to be the most important [23]. At a pH of 6.7 and 1 mM vanadate, the dimer (V2) of V(V) is the only form detected in the spectrum, which does not exclude the presence of the monomeric (V1) form, which should appear at around −558 p.p.m. The latter may be lost in the noise due to the low concentration. The intensity of the tetramer (V4) signal in the spectra (−576 p.p.m.) increases with applied concentration. Since pH determines the degree of protonation of V(V) in the solution, the pH of solutions containing V(V) was kept constant, as it would otherwise affect the chemical shifts of V1 and V2 in ^51^V NMR spectra, while the position of V4 is independent of solution pH [12]. Therefore, the spectra recorded with vanadate in the concentration range between 1 and 10 mM are characterized by identical chemical shifts for all detected vanadate species. However, apart from the expected V2 (−572 p.p.m.) and V4 (−576 p.p.m.), a signal at −574 p.p.m. is detected in the spectra, and its intensity increases with increasing vanadate concentration (Fig. 1b). According to the literature [24], this signal may arise from an interaction between V1 and phosphates, which may further explain the absence of V1 signal. Phosphates have a strong vanadate binding capacity, with a formation constant one order higher than diphosphates [12], and this interaction is most probable. The spectra of vanadate in the medium were recorded due to possible interactions with its components – glucose, asparagine, phosphates, sulphates and microelements. Interactions with glucose and asparagine were not detected by ^51^V NMR in our previous study [25], and the concentration of microelements is too low for detectable interactions.

EPR spectra of the medium with vanadate were also recorded, and no reduction of V(V) to V(IV) was detected (results not shown).

### V and P uptake by *P. blakesleeanus* mycelia

**Fig. 2.**
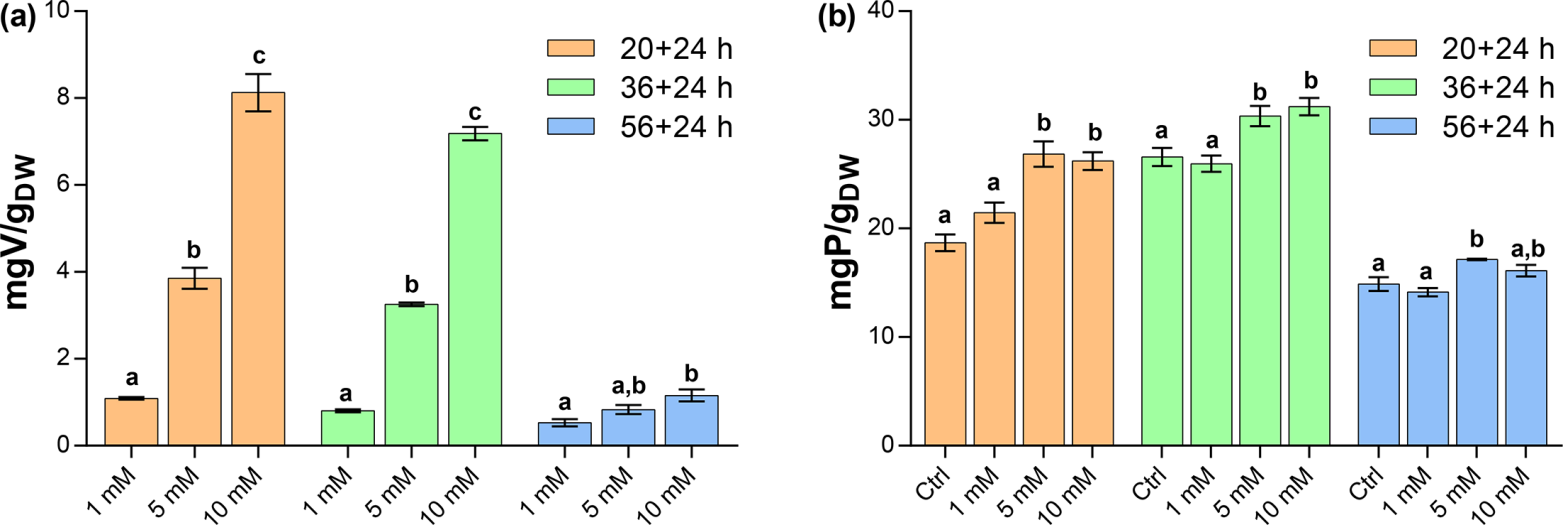
Concentrations of (a) vanadium and (b) phosphorus in mycelia at three different growth stages of the fungus *P. blakesleeanus* (20+24, 36+24 and 56+24 h) after 24 h of treatment with different concentrations of V(V); in a – the controls are not shown as they did not contain vanadate. Values are expressed as mean±se, *n*=3. Statistical significance of differences was analysed using one-way ANOVA with a 0.05 significance level. Pairwise comparisons were made with the Holm-Sidak test.

ICP analysis was used to determine the uptake capacity of *P. blakesleeanus* towards vanadium, and the intracellular content of phosphorus was also analysed, as it is known that the vanadate anion is a phosphate analogue and, as a monomer, is most likely transported by the phosphate transporters [26,27]. Fig. 2(a) shows the vanadium content in the mycelium of *P. blakesleeanus* treated for 24 h at different growth stages. As expected, increasing vanadate concentrations in the growth medium leads to an increased content of V in the fungal biomass. At the mid-exponential and late exponential phases, vanadium content reaches almost 1% of the dry biomass (0.8 and 0.7%, respectively) with 10 mM treatment, while in the stationary phase, maximal V content is 0.1%, which can be explained by the reduced metabolic activity of the mycelium in the stationary phase. This further confirms that the mycelium internalizes V and that this process is energy dependent. Some fungi of the genus *Amanita* are known to be hyperaccumulators of vanadium in the form of amavadin [28]. In *Amanita muscaria*, vanadium content can reach up to 5% of the dry weight [3]. Interestingly, higher concentrations of V(V) – 5 and 10 mM – led to increased phosphate uptake in mid- and late-exponential phase mycelium (Fig. 2b). No phosphorus was found in the supernatant of the respective samples (results not shown). This may seem counterintuitive, as it is assumed that V(V) and Pi use the same transport system and should, therefore, be competitors. However, several studies have found that plants adopt more Pi as V(V) concentration increases, until it begins to exert toxic effects [29,31]. Previous studies with V(V) and *P. blakesleeanus* [16,18] showed fast and considerable effects of V(V) on intracellular phosphorylated compounds, such as an increase in polyphosphates and phosphorylated sugars. However, those experiments had to be performed in a phosphate-free environment to avoid an overlap of extra- and intracellular signals. So there, V(V) caused *de novo* synthesis of polyphosphates from already present phosphates, which was additionally confirmed by a distinct decrease in the intracellular Pi signal. A similar effect of vanadate was demonstrated on the yeast cells of *Hansenula polymorpha* [32]. This strongly implies that the concentration of free phosphate in the cytoplasm becomes lower than required for cellular functions, which is a signal for the additional uptake of phosphate from the surrounding medium.

### V(V) induced ROS generation in *P. blakesleeanus* mycelia

**Fig. 3.**
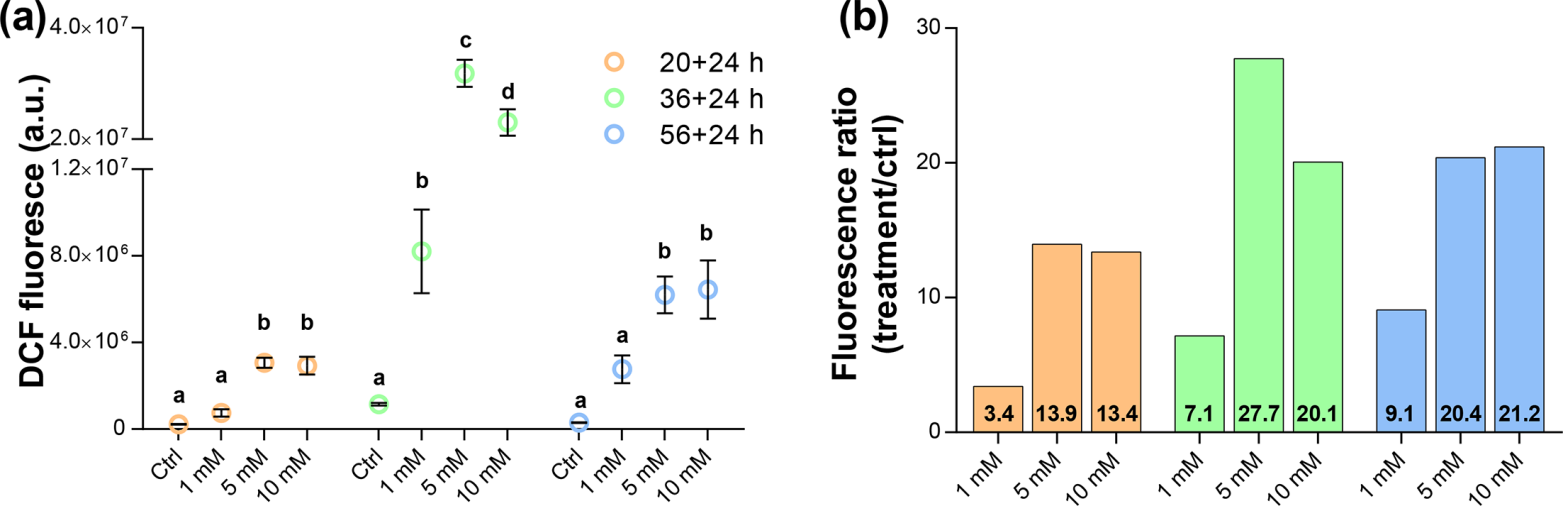
ROS in mycelia at three different growth stages of the fungus *P. blakesleeanus* (20+24, 36+24 and 56+24 h) after 24 h of treatment with different concentrations of V(V). (**a**) Values are presented as a means of DCF fluorescence intensity of the sample±se, *n*=6. Statistical significance of differences was analysed using one-way ANOVA with a 0.05 significance level. Pairwise comparisons were made with the Holm-Sidak test. (**b**) Ratios of ROS in treatments and corresponding controls.

Vanadium is involved in both the generation and annihilation of ROS [33]. ROS concentration was monitored by DCFH-DA assay after 24 h V(V) exposure. Fig. 3(a) shows that the ROS concentrations induced by 5 and 10 mM V(V) are an order of magnitude higher in the 36-h-old mycelium than in the other two age groups. However, the fluorescence ratios (Fig. 3b) show that the increase is not so drastic when compared to the appropriate control. This can be attributed to higher ROS levels in the untreated mycelium itself. In our previous work [19], control ROS levels in 56-h-old mycelia (similar age to 36+24 h) were significantly higher than in younger growth stages. In this context, it should be noted that the 36-h-old control was already 60 h old at the time of testing and thus already in the stationary phase, so the measured ROS concentration could be a cumulative effect of both vanadium and oxidative stress caused by ageing of the mycelium [34]. In fact, the late exponential phase is the only growth phase of the *P. blakesleeanus* mycelium in which V has an adverse effect on growth [19]. On the other hand, basic ROS levels are about four times lower in 56-h-old mycelium (actual age 80 h) than in 36-h-old mycelium, although one would expect a different result based on previous statements. It is possible that the metabolic activity of the mycelium is so low at this late stationary stage, which is reflected in the ROS content. This would also explain a very low uptake of V(V) in this growth phase. The lowest ROS production in the exponential phase mycelium can be attributed to efficient antioxidant activity, while this activity in the late exponential phase mycelium is even lower than in the stationary phase [19], hence the highest ROS production.

### Content of lipids, proteins and carbohydrates changes during growth, and with V(V) treatment

**Fig. 4.**
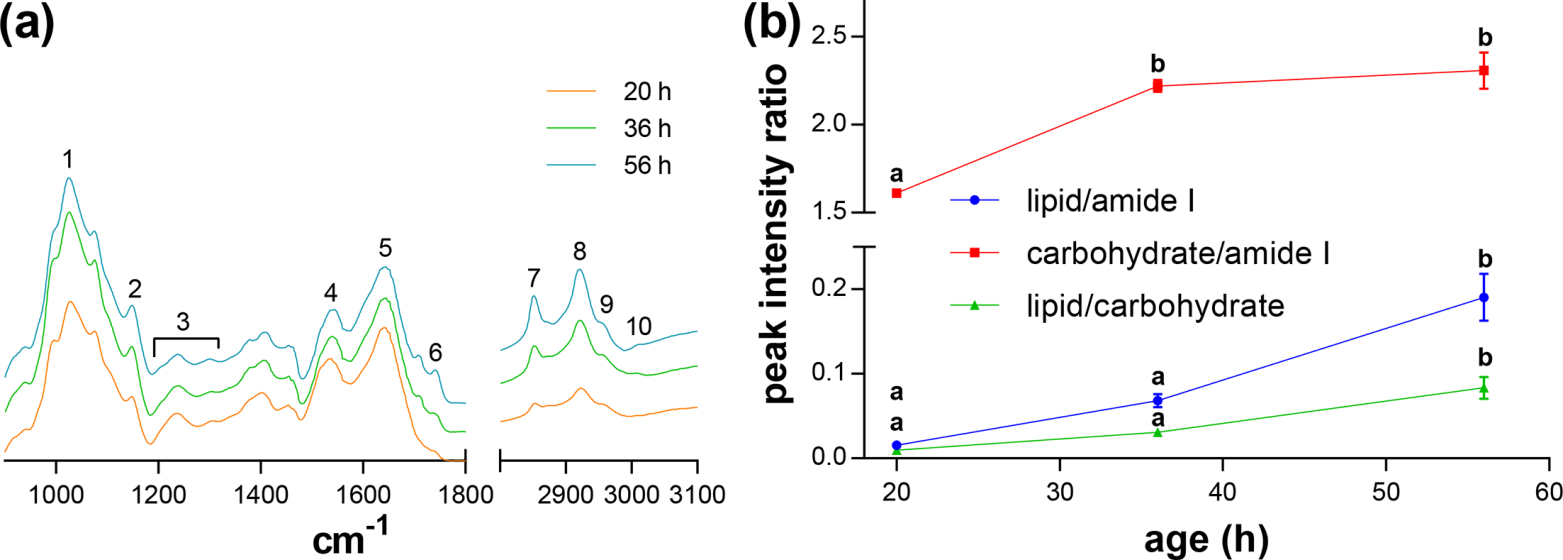
(**a**) Averaged FTIR spectra of controls at 20, 36 and 56 h of growth. The most important peaks are marked and assigned in Table 1. (**b**) Ratios of peaks for lipid content (1,743 cm^−1^), amide I (1,645 cm^−1^) and carbohydrates (1,027 cm^−1^) were used for semi-quantitative analysis of changes in the content of these biomolecules with age and treatment. Statistical significance of differences was analysed using one-way ANOVA with a 0.05 significance level (*n*≥3). Pairwise comparisons were made with the Holm-Sidak test.

FTIR spectroscopy has already been successfully applied as rapid and non-invasive technology for the fingerprinting of cellular chemical components [15]. It has been used to identify different strains of the dry rot fungus *Serpula lacrymans* [35], for high-throughput screening of lipid production [36] and other high-value metabolites in Mucoromycota fungi [15], and for changes in cellular biomolecules in response to nutrient stress in microalgae [37]. Fig. 4(a) shows averaged spectra of *P. blakesleeanus* controls in three selected growth phases with marked peaks of specific functional groups. Peaks were assigned according to Movasaghi *et al.* [38], Malek *et al.* [39], Kosa *et al.* [36], Dzurendova *et al.* [15], Shapaval *et al.* [40] and Al-Kelani and Buthelezi [41] (Table 1). The spectral intensities depend on sample morphology and chemistry [42] as well as homogeneity and therefore cannot be considered a quantitative method under all conditions of acquisition and sample preparation. However, the ratio of peak intensities within the spectrum may indicate changes in the proportions of biomolecules in the biomass either during ageing or after treatment, and in this sense, FTIR can be considered semi-quantitative. For this analysis, the band at 1,743 cm^−1^ associated with C=O stretching of carbonyl compounds [37,38, 43], the amide I band at 1,645 cm^−1^ [38,41] and the region between 1,180 and 950 cm^−1^ were used to analyse the lipid, protein and carbohydrate content, respectively. Fig. 4(b) shows lipid/amide I, lipid/carbohydrate and carbohydrate/amide I ratios at three selected growth stages without treatment. Compared to 20-h-old mycelium, the lipid/amide I ratio increases 4.4× in 36-h-old mycelium and 12.3× in 56-h-old mycelium. Lipids in fungi are major constituents of membranes and cell walls and serve as carbon and energy storage in the form of lipid bodies [44]. With a lipid content of 20–28% [45], *P. blakesleanus* can be regarded as an oleaginous fungus. According to Cerda-Olmedo and Avalos [46], the lipid content in *P. blakesleanus* can reach up to 41% of dry weight in a neutral medium, which was used in this study, as the conversion of sugars to lipids is highest under these conditions. In addition, nitrogen starvation, inevitable in an ageing batch culture, is known to increase lipid content in many micro-organisms, and *P. blakesleeanus* has been shown to increase the number of lipid droplets under these conditions [47]. Carbohydrates/amide I ratio increases only slightly, 1.3× and 1.4× in 36- and 56-h-old mycelium, respectively, compared to 20-h-old mycelium, while the lipid/carbohydrate ratio increases 3.2× and 8.7×, respectively. The proportion of lipids in the biomass obviously increases the most with the age of the culture compared to proteins and carbohydrates, suggesting that the nitrogen source in the medium is depleted faster than the carbon source, which is required for the synthesis of fatty acids [48].

**Table 1.** Peak assignment of spectra depicted in Fig. 4(a)

Peak no.	Wavenumber (cm^−1^)	Peak assignment	Biomolecules
**1**	1,028	C-O and C-C stretching; C-O-H deformation motions	Carbohydrates, glycogen
**2**	1,149	C-O stretching	Glycogen, mucin
**3**	1,190–1,330	Amide III region	–
**4**	1,520–1,580	Amide II region	Proteins, chitin
**5**	1,580–1,720	Amide I region	Proteins
**6**	1,743	C=O stretching of carbonyl compounds	Lipids
**7**	2,850	-CH_2_ symmetric stretching	Lipids
**8**	2,920	-CH_2_ asymmetric stretching	Lipids
**9**	2,954	CH_3_ asymmetric stretching (C-H)	Lipids
**10**	3,008	=C-H stretching of unsaturated lipids	Lipids

**Fig. 5.**
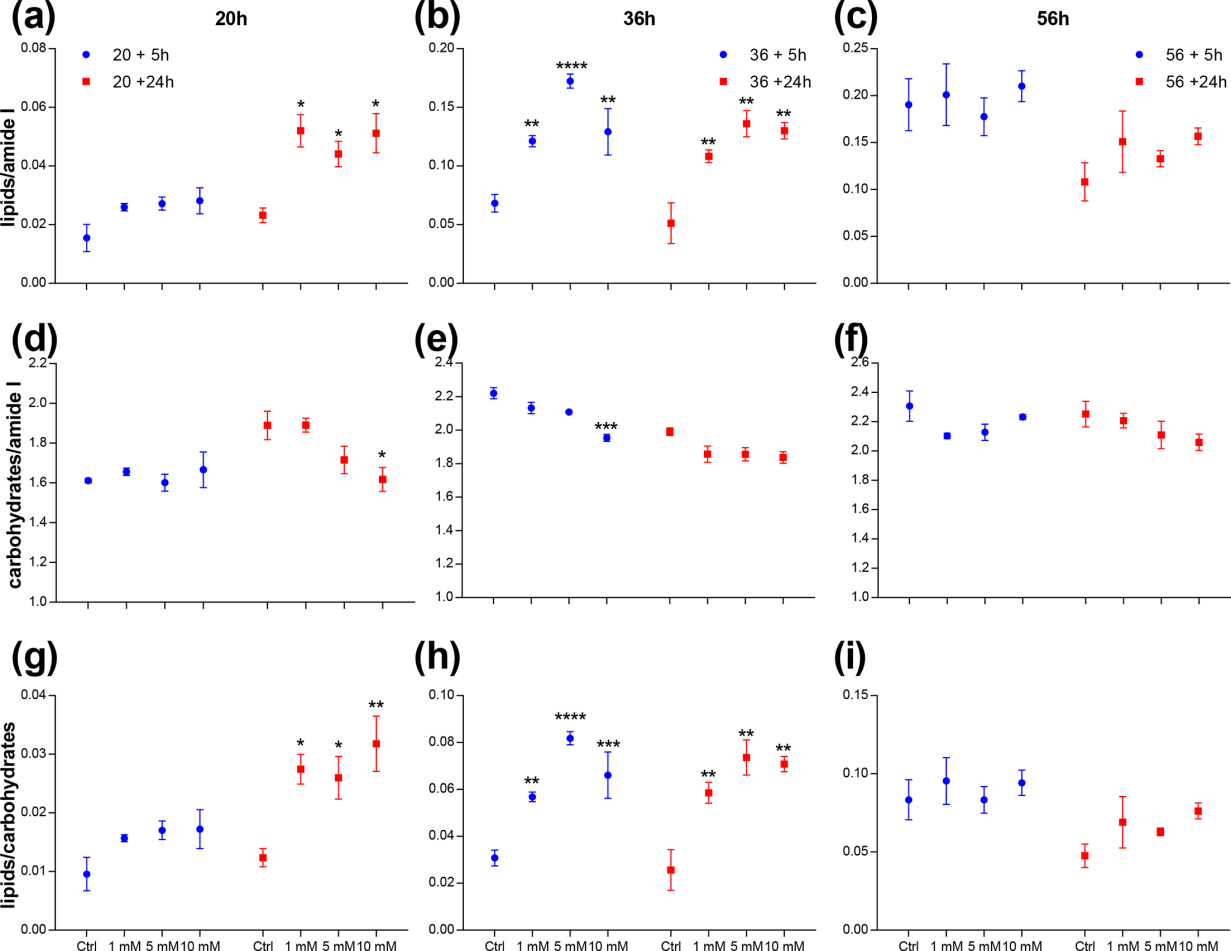
The effect of treatment with increasing concentrations of vanadate (1, 5 and 10 mM) applied in three different stages of growth (20, 36 and 56 h) on the ratios of lipids, proteins and carbohydrates. The effect was determined after 5 and 24 h of treatment. Statistical significance of differences was analysed using one-way ANOVA with a 0.05 significance level (*n*≥3). Comparisons to control were made with the Holm-Sidak test.

The effects of 1, 5 and 10 mM V(V) were investigated in all three growth stages after 5 and 24 h of incubation (Fig. 5). At the stationary stage of the mycelium (Fig. 5c, f, i), no significant changes occurred with any of the treatments. This is at least partly due to the lowest V(V) uptake in this growth stage (Fig. 2a) and low metabolic activity of the ageing culture. In 20-h-old fungi, the lipid portion of biomass increases in relation to both proteins and carbohydrates after 24 h of treatment with all applied V(V) treatments, while in 36-h-old mycelium, it increases already after 5 h of treatment. Fungal lipids may be suitable for biodiesel production or resemble highly nutritious and valuable oils with a high content of polyunsaturated fatty acids [15]. *P. blakesleeanus* belongs to the division of Mucoromycota, in which many strains produce a high content of lipids [49], accompanied by other high-value metabolites such as pigments, polyphosphates and chitosan [50]. According to Chenouda [45], oleic acid, linoleic acid, linolenic acid, stearic acid and palmitic acid are the major fatty acids present in *P. blakesleeanus*, but to the best of our knowledge, there have been no attempts to establish commercially sustainable lipid production with this strain. In contrast to the mycelium, the role of lipid globules in stage-1 sporangiophores has attracted more interest, and it has been established that they act as gravisusceptors of the sporangiophore with buoyancy as the physical principle for their mode of action [51]. Further studies are needed to establish whether vanadate treatment alters the fatty acid composition. The commercial value of this potential production could be increased by the possibility of simultaneous isolation of polyphosphates or chitosan, one of the basic constituents of the cell wall of this strain [52]. Previous studies have shown [18] that vanadate increases polyphosphate content in *P. blakesleeanus*, and we have shown here (Fig. 2b) additional uptake of phosphates from the medium upon vanadate addition.

Regarding the carbohydrate/amide I ratio (Fig. 5d–f), the V(V) treatment has a slightly decreasing effect. The analysed region (1,180–950 cm^−1^) indicates the presence of cell wall carbohydrates [53,54], but also the storage carbohydrates such as glycogen [38,41], a major storage carbohydrate in *P. blakesleeanus* [55]. A short-term effect of V(V), but not V(IV), on *P. blakesleeanus* is an increase in the level of phosphorylated sugars, primarily glucose-1-phosphate (G1P), that could arise from glycogen degradation [18], but it is not known whether this effect persists after 24 h of incubation. On the other hand, stress conditions such as an increase in ROS content (Fig. 3) could lead to modifications in cell wall composition and metabolic rates, resulting in a weakening of the cell walls [3,54].

**Fig. 6.**
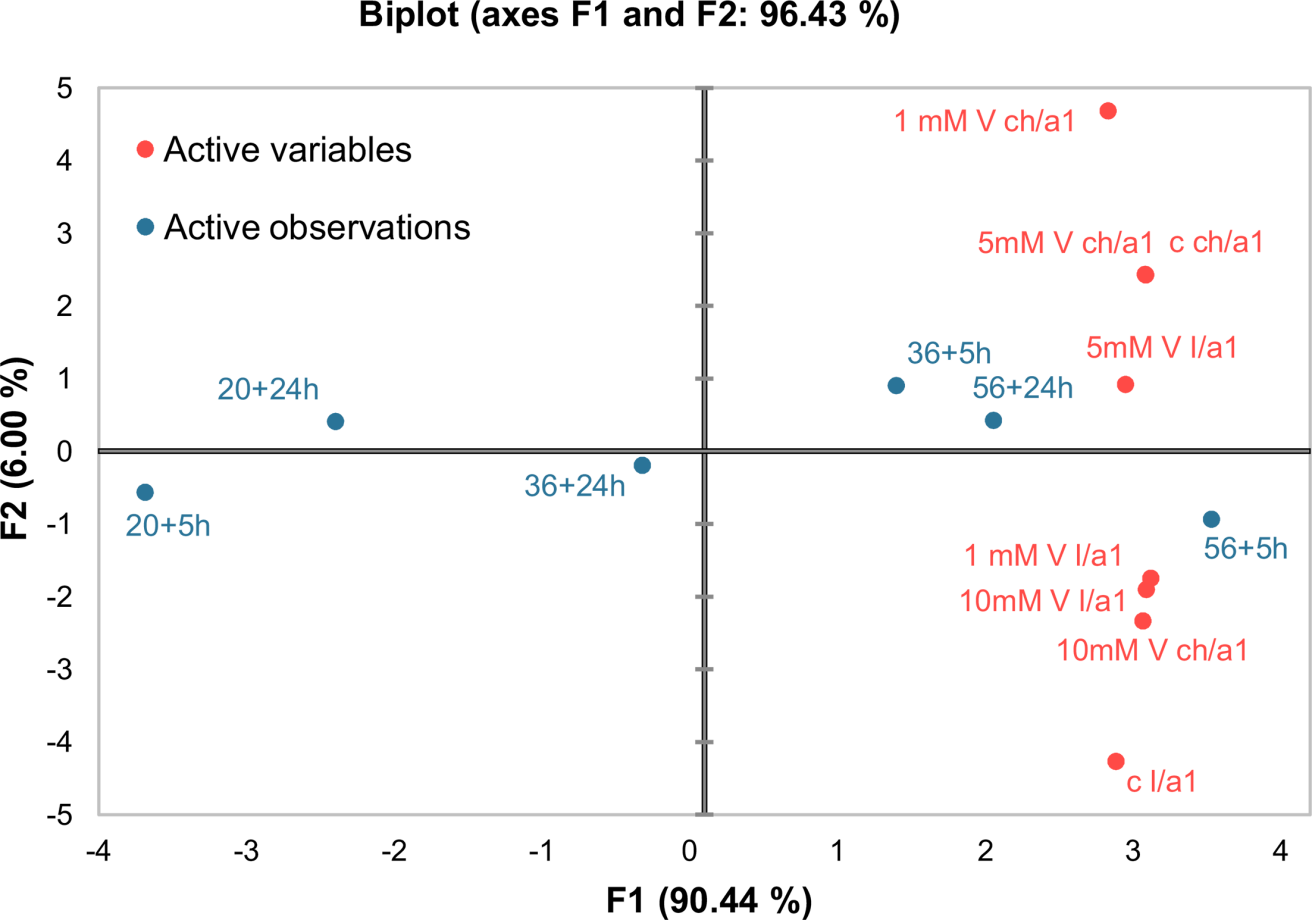
Correlation matrix PCA biplot of the biomolecule content in control (**c**) and V(V) treated samples (for parameter explanation, see Fig. 5). Differentiation along the F1 axis accounts for 90.44% variation and relates mainly to the mycelia developmental stage. Differentiation along the F2 axis accounts for 6% variability and can be ascribed to vanadate treatment.

To summarize the interrelations of growth phase and V(V) effects on lipid, protein and carbohydrate content, a correlation matrix PCA was performed. The results are presented as a biplot to relate the effects of growth length and V(V) supplementation (Fig. 6). Due to the similarity in the lipid/amide I and lipid/carbohydrate ratios, the latter was omitted for clarity. In the course of the analysis, two factorial axes accounting for 90.44% (F1) and 6.00% (F2) of total inertia were retained.

Analysis of the PCA plane shows that the growth stages are aligned almost in a single file along the F1 axis, starting with the mycelium at the mid-exponential stage (20 h old) at the negative end and ending with the mycelium at the stationary stage (56 h old) at the positive end (Fig. 6). This indicates that the content of both carbohydrates and lipids in the mycelium increases steadily during development. Therefore, the F1 axis can be referred to as the developmental axis. Given that the F1 axis accounts for the majority of variance, it is evident that developmental changes have a much more significant influence on the content of carbohydrates and lipids in the mycelium compared to the V(V) treatment. V(V) treatments are well distributed along the F2 axis; consequently, it can be referred to as the V(V) axis (Fig. 6). Although V(V) effects are much less pronounced than developmental ones, they are still very clear since control samples are separated from the V(V) treated samples along the F2 axis. In the case of lipid content, this holds true for all vanadate concentrations, while for carbohydrates, it is only valid at the highest V(V) concentration (10 mM). The PCA analysis also indicates that vanadate treatment has opposite effects on the content of lipids and carbohydrates in the mycelium, increasing the former and decreasing the latter.

## Conclusions

*P. blakesleeanus* is a fungus whose physiology has been abundantly studied, especially regarding the sporangiophores. The mycelium itself has attracted somewhat less interest, although its fast growth and short life cycle provide the opportunity to quickly and reliably test interactions with various environmental factors and uncover potential industrial applications. This study showed that *P. blakesleeanus* mycelium in the mid-exponential phase can take up V(V) to almost 1% of dry weight, with simultaneous increased Pi uptake. As V is a redox-active element, it increases ROS formation in the mycelium, which still shows a high tolerance, primarily due to the activation of its antioxidant systems. Tolerance can partly be attributed to the increased intake of phosphates, which have the role of chelators, while also ensuring the functioning of enzymes whose function depends on phosphorylation. In addition, V(V) significantly increases the proportion of lipids compared to proteins and carbohydrates in both the mid- and late-exponential phases.

Since *P. blakesleeanus* is not a soil fungus, it cannot be considered a factor for bioremediation but is a plausible candidate for V bioaccumulation. This study opens the door to at least two lines of applicative research. The potential for V(V) bioaccumulation is important, bearing in mind the anthropogenic vanadium increase in the environment. More extensive studies are needed that involve monitoring the uptake potential of this fungus over several growth cycles and in the presence of waters with elevated vanadium content. On the other hand, the increase in lipid content after V(V) treatment calls for an in-depth study of fatty acid composition, potential industrial utilization and possibilities for large-scale production.

## Abbreviations

DCF: dichlorofluorescein
DCFH-DA: dichlorodihydrofluorescein diacetate
FTIR: Fourier Transform Infrared
PCA: principal component analysis
ROS: reactive oxygen species
